# Poultry red mite (*Dermanyssus gallinae*) infestation: a broad impact parasitological disease that still remains a significant challenge for the egg-laying industry in Europe

**DOI:** 10.1186/s13071-017-2292-4

**Published:** 2017-08-01

**Authors:** Annie Sigognault Flochlay, Emmanuel Thomas, Olivier Sparagano

**Affiliations:** 1 Merck Animal Health, 2 Giralda Farms, Madison, NJ 07940 USA; 2 MSD Animal Health Innovation GmbH, Zur Propstei 55270, Schwabenheim, Germany; 3 Coventry University, Vice-Chancellor Office, Alan Berry Building, Coventry, CV1 5FB UK

**Keywords:** Poultry red mite, *Dermanyssus gallinae*, Ectoparasite, Acaricide, Zoonosis, One health, Occupational safety, *Salmonella*, Vector, Drug resistance

## Abstract

The poultry red mite, *Dermanyssus gallinae*, has been described for decades as a threat to the egg production industry, posing serious animal health and welfare concerns, adversely affecting productivity, and impacting public health. Research activities dedicated to controlling this parasite have increased significantly. Their veterinary and human medical impact, more particularly their role as a disease vector, is better understood. Nevertheless, red mite infestation remains a serious concern, particularly in Europe, where the prevalence of red mites is expected to increase, as a result of recent hen husbandry legislation changes, increased acaricide resistance, climate warming, and the lack of a sustainable approach to control infestations. The main objective of the current work was to review the factors contributing to this growing threat and to discuss their recent development in Europe. We conclude that effective and sustainable treatment approach to control poultry red mite infestation is urgently required, included integrated pest management.

## Introduction

It is well established that the poultry red mite, *Dermanyssus gallinae* (De Geer, 1778), is the most damaging parasite of laying hens worldwide. The impact of red mite infestation in Europe has been thoroughly described in scientific literature, for over 20 years. Red mite infestations pose serious animal health, welfare and public health concerns, and affect the productivity of the egg industry [1–6]. Access to effective and safe medical treatments has been an unmet need. This review describes the factors contributing to this omnipresent impact and discusses their recent development in Europe.

## Poultry red mite infestation poses increasing animal health and welfare concerns

### Prevalence

The first source of concerns associated with red mite infestation is the extremely high and increasing prevalence of this disease in Europe. A recent epidemiological review reports that 83% of the European farms are infested by *D. gallinae*. This prevalence reaches 94% in The Netherlands, Germany and Belgium [1]. Poultry red mite infestation affects all production types, from backyard or organic farms, to more intensive, enriched cage or barn systems [2]. The impact of poultry red mite infestation has been increasing in Europe for the past decades and is expected to further increase.

One of the first factors contributing to this increase is the recent transformation of housing systems in laying hen husbandry in EU member countries. Directive 1999/74/EC on egg production and egg trade has banned the use of traditional cages for poultry birds since 2012. Although designed to improve the welfare of laying hens, this legislation has resulted in the move to housing systems incorporating more complex environments which appear to favor mite proliferation and exacerbate the problem of red mite infestation [7–10]. For instance, enriched cages give far more hiding places for red mites to escape effective treatments. Mite infestation rates have been described to be much lower in hens kept in traditional cage systems compared to alternative ones [10, 11]. In 2009, before the first banning of conventional cages (Austria and Germany prohibited such cages from 2010 onwards), 74.4% of the laying hen housing systems still consisted in conventional cages in the European Union. In 2013, all member states had been able to complete the transformation process from conventional cages to mainly enriched cages, barn systems, and free range housing systems [12], meaning that within four years after 2009, the high majority of laying hens was transferred from a system unfavorable to mite proliferation to a system favoring it.

Another environmental factor expected to favor the proliferation of red mite infestation in the future is climate warming. During extreme weather events, red mite increased populations have been implicated in the deaths of large numbers of hens during the summer heat wave of 2003 [13].

Finally, the removal of several acaricide products from national markets due of safety concerns and the sustained lack of new effective control methods may have aggravated the *D. gallinae* prevalence in Europe. This factor is further described later in this review.

### Clinical effects of mite infestation

In addition to the high prevalence of the disease, another concern is the severity of the effects induced by *D. gallinae* parasitism on the birds’ health and welfare. The first clinical sign observed in infested animals is sub-acute anemia due to repeated mite bites. A laying hen can lose more than 3% of its blood volume every night [3]. In extreme cases, *D. gallinae* infestation burdens may be so heavy that hens may die from severe anemia [14–16]. Two reports, detailing the effects of heavy mite infestations in layer farms in Poland and Romania, describe a 6.2% and a 10-fold increase in hen mortality due to red mite infestations [14, 15].

### Disease vector role of *D. gallinae*

Besides this direct effect of hematophagous parasitism, *D. gallinae* has also been implicated as a vector for a number of avian viral and bacterial pathogens of animals and humans. These include the paramyxovirus that causes Newcastle disease, the Eastern, Western, and Venezuelan equine encephalomyelitis viruses, and bacteria such as *Escherichia coli*, *Erysipelothrix rhusiopathiae*, *Pasteurella multocida*, *Salmonella gallinarum* and *S. enteritidis* and avian influenza A virus [5, 6, 17–22].

Poultry mites often serve as a long-term host of viral and bacterial pathogens, thus becoming a reservoir for these agents and exacerbating the vector potential of *D. gallinae*. For example, eastern equine encephalomyelitis virus and *P. multocida* were isolated from mites 30 days and two months, respectively, after ingestion of blood meals from infected chickens [21]. The ability of the mite to survive between successive flocks and its persistence in a fasting state for extended periods of time enhance its vectorial role in maintaining pathogen agents on poultry farms [23].

### Impact of mite infestation on bird’s welfare

First, the presence of mites in a production house induces a high level of stress in the birds. Stress is induced by pain and skin irritation associated with repeated mite bites favored by the very high parasite load typical of red mite infestations, with mite densities ranging from 25,000 to 500,000 mites per hen [1, 3, 24]. In addition, mite infestations induce aggressive feather-pecking and cannibalistic behavior, increased feed and water intake, and decrease general animal health [3, 4, 25, 26]. Higher noise volumes are typically observed by farmers in mite infested houses. Increased self-grooming, a characteristic symptom of anxiety, is observed in artificially infested hens [24]. The severity of injuries resulting from such behavior is currently limited by beak-trimming, but is expected to increase following the scheduled ban on beak trimming across several European member states in 2016 [1]. Kowalski & Sokol [27] showed that mite infestation led to a 1.5-fold increase in corticosterone blood levels and a 22% decrease in β-globulin levels, indicating somatic stress and immunosuppression. The adrenaline levels were also more than twice as high as in the control animals, indicating psychogenic stress. For all these reasons, poultry red mites infestation is widely recognized as an animal welfare issue by the scientific community [7], and was a major topic at the June 2009 European Symposium on Poultry Welfare [28].

## Growing impact of red mite infestation on public health

In addition to its effects on chicken’s health and welfare, red mite infestation also poses public health concerns, due to the role of *D. gallinae* as a disease vector of zoonotic diseases, and its medical impact on humans living or working in close association with poultry.

### Role of *D. gallinae* in transmission of zoonotic diseases

As described above, *D. gallinae* is involved in the transmission of numerous poultry pathogens, including zoonotic pathogens like *Salmonella enteritidis* [17–19, 26], which is responsible for one of the most widespread zoonoses worldwide, non-typhoidal salmonellosis. This disease has the highest global human mortality rate of any zoonotic disease, with most cases being of food-borne origin, and poultry products being one of the most common sources of the disease [18, 20]. Mites become carriers of *Salmonella* either by external cuticular contact or ingestion of a blood meal from infected birds [19]. *Salmonella* has been found to survive internally in *D. gallinae* for up to four months [21], with bacterial reproduction occurring within the mites [19]. *D. gallinae* may transmit *Salmonella* to poultry when birds orally ingest infected mites [17, 19].

*Borrelia burgdorferi*, the causative agent of Lyme disease, and avian influenza A virus, mentioned above as part of the avian pathogens, have been recently added to the list of zoonotic pathogens potentially transmitted by *D. gallinae* [6, 22].

### Medical impact of red mite infestation

Red mites are of growing concern in human medicine. *D. gallinae* infestation is increasingly responsible for human dermatological lesions, namely gamasoidosis, particularly in people living or working in close proximity to poultry [5]. A recent survey reported an increasing incidence of gamasoidosis worldwide, and that the disease is underdiagnosed [6]. The survey showed that the severity of the disease is exacerbated by the persistency of the infestation, the frequent treatment failures, and, as described above, the potential transmission of zoonotic diseases by the mites, such as *Borrelia burgdorferi*, *Babesia* and *Bartonella*. Complete prevalence data on gamasoidosis in poultry workers are not available. However, the 19% incidence of contact dermatitis reported in a two-year survey of workers on 58 European poultry farms is probably a reasonable indication of occupational risk [29]. Many gamasoidosis cases are misdiagnosed or go unreported [30], suggesting that actual incidence is higher than commonly assumed.

Poultry red mite infestation is therefore definitely a matter for the “One Health” initiative [31], an approach that considers both veterinary and human health implications of mite infestation which is one of the central working areas the European Cooperation in Science and Technology (COST) conference for sustainable Control of the poultry Red Mite (COREMI, http://www.coremi.eu/home.html) [32]. In 2011, a group of European researchers in this field claimed that they “wholeheartedly support the inclusion of the red mite as a zoonotic agent in all regulations regarding occupational safety, and poultry red mite infestation as an occupational hazard for individuals working with poultry” [29].

## Productivity losses due to red mite infestation have significantly increased

Economic losses from poultry mite infestation severely affect the productivity of the egg industry. Consequences of red mite infestation in a layer operation include primarily a negative impact on feed conversion ratio, a drop in egg production, an increase in downgraded eggs, a higher susceptibility to poultry diseases, and more dead animals [1]. A still widely quoted estimate for the cost of mite control and production losses is €130 million annually [3]. Because this commonly used number was calculated in 2005, it underestimates the cost of red mite infestation in Europe at the present time. First, the laying hens population, estimated at 350 million heads in 2005 [3], has increased significantly. In 2013–2014, the Statistics Division of the Food and Agricultural Organization of the United Nations has estimated the number of layer chickens in the 17 largest egg-producing countries in Europe to be 431 million [33]. Second, the high infestation rate in European farms has become an increasingly important concern. Several European countries have recently reported prevalence rates of more than 90% [1], versus 80% for the most affected countries about a decade ago [3, 8].

Van Emous (2005) [3] estimated the impact of mite infestations on productivity to be €43/hen, including €0.14 for mite treatment (direct costs), and €0.29 for productivity losses (indirect costs). The estimation of the evolution of direct treatment costs is complex due to the changes in the acaricidal treatments arsenal available to farmers in the past decades, as described later in this review. Recently, in 2017, the same author [34] estimated that the current total cost of red mite infestation is €0.60 per hen per year in the Netherlands (€0.15 for direct costs, and €0.45 for productivity losses), which represents an increase of about 40% compared to 2005 for the total cost of mite control per layer head. Overall, the damage caused by mites in Europe is now estimated at about € 231 million [34]. Van Emous explains this higher damage by the conversion of traditional cages to alternative housing systems, the longer production life-cycles of the animals, and the ban of beak trimming [34].

## Control of mite infestations remains a major unmet medical need

The increased need for a sustainable approach to control poultry mite infestation has been thoroughly described. A very limited number of chemical treatments are currently available to treat mite infestations [2, 35]. Many conventional mite products have been withdrawn from European markets or banned in the past few years because they did not comply with European or national regulatory requirements for human consumer and user safety. The main product classes affected were carbamates (carbaryl, methomyl, propoxur), organophosphates (dichlorvos, fenitrothion, chlorpyrifos, diazinon), and pyrethroids (cyhalothrin). At the time of writing, the organophosphate phoxim (Byemite®, Bayer [36]) is the only veterinary medicinal product registered in Europe for the treatment of *D. gallinae* infestations (since 2010). However, it is not licensed in countries with the largest layer industries like Germany, Poland, Spain and the UK, where the prevalence of *D. gallinae* infestations exceeds 80% [1, 3, 8]. Although this spray treatment is allowed for application in the presence of birds in the infested house, it should not be sprayed onto the birds. This required precaution of use may prevent the active compound to reach mites hidden in the refuges located very close to the birds. Furthermore, an egg withdrawal period of twelve hours has to be observed after treatment, which makes this product unsuitable for use in large caged layer farms. Finally, the use of organophosphates as a solution to control mite infestation is limited by the widespread resistance of *D. gallinae* to this class of acaricide [35, 37].

Besides phoxim, several acaricidal spray products are available in some European countries, mainly for use during the unoccupied cleaning period between two flocks, for the treatment of the poultry house and equipment. For example, pyrethroids (cypermethrin, permethrin, deltamethrin), carbamates (bendiocarb), abamectin and spinosad are available as formulations for spray application. Some of these products have no recommended egg withdrawal time, which poses a serious human food safety risk if used off label, in the presence of birds. Only a few compounds*,* e.g. spinosad (Elector®) and cypermethrin (Intermitox®) in Germany, can be applied in the presence of birds. Misuse or even illegal use of acaricidal compounds (e.g. amitraz, fipronil, ivermectin, diazinon, carbaryl, and other pesticides used in agriculture) for the treatment of *D. gallinae* in poultry houses are suspicious of common use in certain areas. This poses critical risks to consumer safety, but is also a reason for resistance development as a result of underdosing [38–41]. A recent survey in Poland revealed that 50% of the 32 enrolled laying farms use products with “unknown ingredients” to treat *D. gallinae* infestation [37].

**Fig. 1:**
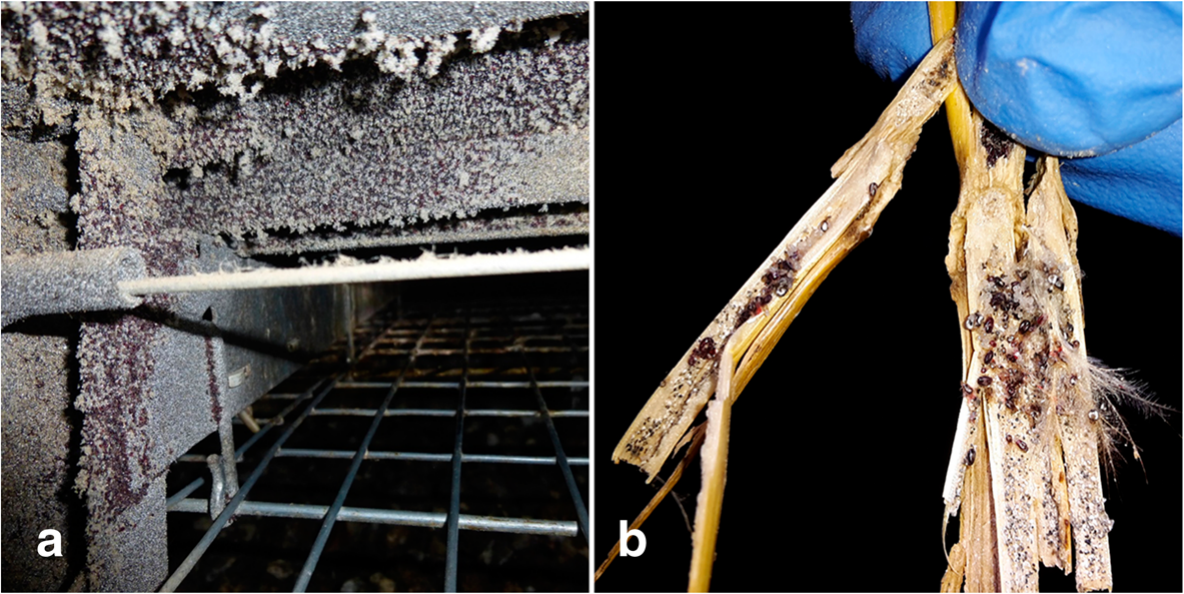
Environmental infestation with poultry red mites. **a** Red mite cluster on the ceiling of a cage at a laying farm. **b** Red mites and mite eggs hidden in straw litter from a laying-hen building. High infestation densities make it difficult to successfully control *Dermanyssus gallinae* using environmental control alone

Successful chemical treatment is also hampered by resistance development to multiple acaricides [4, 42] due to creation of resistant mites as a result of improper treatment application [35, 36]. Uneven spraying, especially inside crevices and cracks or litter (Fig. 1) may lead to exposure of mites to sublethal concentrations. Additionally, currently marketed acaricidal products have only short residual activity [43], which is a problem when targeting *D. gallinae* mites that may not encounter treated surfaces until several days after application. Furthermore, these products are applied only once, and are either not substantially active or inactive on mite eggs, so eggs develop into further stages, enabling regrowth of mite infestation burdens in the poultry houses.

Some non-chemical methods of control are used, but none has a satisfactory risk-benefit balance. Although silica-based products are widely used, their purity and the size of their particles vary greatly between products, and they pose serious safety threats for user and animal due to the irritation of the respiratory tract caused by inhaled silica particles, which justified the recent ban of these products in the Netherlands. Natural acaricides, including essential oils or plant-derivatives may have variable concentrations as active ingredients and may be harmful to humans and animals [26]. Predator mites have not shown satisfactory efficacy so far [2]. The development of new vaccine-based control strategies is a promising approach; a vaccine under development reduced mite counts in infested birds, but not to an adequate extent [44, 45]. Heating of the house up to 60 °C during the unoccupied period has been described as effective; however, this is perceived as expensive and not suitable for farms with plastic equipment components [26]. The use of diesel oil or washing-up liquids to treat mite infestations has also been described [46].

Access to an effective, convenient and safe medical treatment for red mite infestation has been an unmet need for nearly two decades: as early as 1998, the need for a systemic substance was expressed to avoid stressing chickens and uneven spray distribution [4]. Since then, only one veterinary medicinal product against poultry mite infestation has been licensed in a few European countries (phoxim, Byemite®, Bayer). As detailed above, all other solutions currently available are non-chemical products with efficacy not scientifically researched, or chemical sprays with limited value due to their mode of application or the widespread development of resistance.

Several unlicensed (or even banned) products are still widely employed in Europe [2]. A recent survey showed the presence of pesticides banned by the European Union (carbaryl) or not licensed for use on layers (permethrin) in the tissues of laying hens slaughtered for human consumption [41]. This further emphasizes the severe risks that the lack of effective and authorized products pose to human food safety.

This unmet medical need and the urgent need for innovative treatment approaches have clearly been recognized by the scientific community, the layer industry, and the European Union. The absence of an effective treatment is stated in most of the scientific papers and has been recognized by the EU Commission, which created and funds the COST Action FA 1404 Research Platform (COREMI, Control of Red MItes) to “rid laying hens of a common disease-spreading pest”. This four-year project started in December 2014 and involves representatives from almost all European countries, Turkey, and Israel. One of the major conclusions from recent COST conferences was that no single treatment method is sufficient to control *D. gallinae*. Increased use of integrated pest management, improved biosecurity measures to prevent transmission of mites, and mite infestation monitoring are considered the best current methods to control *D. gallinae* infestation. There remains a great demand for developing more useful, effective and innovative treatments to keep red mite infestations under control, including newer generation acaricides [35].

## Conclusion

Since the last reviews on the impact of *D. gallinae* in Europe, research activities dedicated to controlling this parasite has increased significantly. However, poultry red mites remain a significant animal welfare concern and a serious threat to the egg production industry. Their veterinary and human medical impact, more particularly their role as a bacterial and disease vector, is better understood. The significance of poultry red mites in Europe is expected to increase as a result of recent hen husbandry welfare legislation, increased acaricide resistance, and the lack of a sustainable approach to control infestations. Work is urgently required to develop effective and sustainable treatment approach to control poultry mite infestation, included integrated pest management.

## Abbreviations

COREMI: Control of the poultry red mite
COST: European Cooperation in Science and Technology
FAO: Food and Agriculture Organization
